# BacScan: a novel genome-wide strategy for uncovering broadly immunogenic proteins in bacteria

**DOI:** 10.3389/fimmu.2024.1392456

**Published:** 2024-05-08

**Authors:** Junhua Dong, Qian Zhang, Jinyue Yang, Yacan Zhao, Zhuangxia Miao, Siyang Pei, Huan Qin, Changwei Jing, Guoyuan Wen, Anding Zhang, Pan Tao

**Affiliations:** ^1^ State Key Laboratory of Agricultural Microbiology, College of Veterinary Medicine, Huazhong Agricultural University, Wuhan, Hubei, China; ^2^ Key Laboratory of Prevention & Control for African Swine Fever and Other Major Pig Diseases, Ministry of Agriculture and Rural Affairs, Cooperative Innovation Center for Sustainable Pig Production, Huazhong Agricultural University, Wuhan, Hubei, China; ^3^ Hubei Hongshan Lab, Wuhan, Hubei, China; ^4^ College of Life Science, Wuhan University, Wuhan, Hubei, China; ^5^ Institute of Animal Husbandry and Veterinary Sciences, Hubei Academy of Agricultural Sciences, Wuhan, Hubei, China

**Keywords:** bacterial subunit vaccine, vaccine target, immunogenic protein, protective antigen, genome-wide screening, streptococcus suis

## Abstract

In response to the global threat posed by bacterial pathogens, which are the second leading cause of death worldwide, vaccine development is challenged by the diversity of bacterial serotypes and the lack of immunoprotection across serotypes. To address this, we introduce BacScan, a novel genome-wide technology for the rapid discovery of conserved highly immunogenic proteins (HIPs) across serotypes. Using bacterial-specific serum, BacScan combines phage display, immunoprecipitation, and next-generation sequencing to comprehensively identify all the HIPs in a single assay, thereby paving the way for the development of universally protective vaccines. Our validation of this technique with *Streptococcus suis*, a major pathogenic threat, led to the identification of 19 HIPs, eight of which conferred 20-100% protection against *S. suis* challenge in animal models. Remarkably, HIP 8455 induced complete immunity, making it an exemplary vaccine target. BacScan’s adaptability to any bacterial pathogen positions it as a revolutionary tool that can expedite the development of vaccines with broad efficacy, thus playing a critical role in curbing bacterial transmission and slowing the march of antimicrobial resistance.

## 1 Introduction

Bacterial pathogens, notably responsible for 13.6% of global deaths, were the second leading cause of death worldwide in 2019 (1). In addition, the overuse of antibiotics has led to the development of antimicrobial resistance, which has become an emerging threat to public health (2), highlighting the urgency of effective vaccination. However, each bacterial species typically has multiple serotypes, and most bacterial vaccines are less effective against heterologous serotypes (3–5). Therefore, a broadly protective vaccine that provides protection against divergent serotypes within bacterial species is urgently needed to reduce the transmission of bacterial pathogens.

An efficient approach to develop broadly protective vaccines is to use the antigenic proteins that are conserved across different serotypes as vaccine targets. Many genome-wide screening strategies have been developed for antigen discovery, and each has its advantages and disadvantages (6–8). For example, reverse vaccinology uses *in silico* analysis of the bacterial genome to identify typically hundreds of candidate protein antigens, which are heterologously expressed and individually evaluated for vaccine potential in an animal model. This approach was first applied to *Neisseria meningitidis*, and three out of 350 candidate proteins were identified for the development of a subunit vaccine that is now licensed in 30 countries (9, 10). However, it is costly and time-consuming to express hundreds of proteins and evaluate their protective efficacy in animals. Filamentous phage libraries that randomly display bacterial proteins or peptides have been screened with bacterial-positive sera to identify highly immunogenic antigens, which specifically bind to sera and are enriched after multiple rounds of panning (11). However, filamentous phages assemble in the periplasm of *E. coli*, and expressed antigens must be transported to the periplasm to be displayed on phage capsids (12, 13). In addition, the expression of certain exogenous proteins can limit the proliferation of the recombinant phages. As a result, these phages are lost after multiple rounds of amplification in *E. coli*. Therefore, this traditional phage display may have a selection bias. Two-dimensional electrophoresis of *in vitro*-cultured bacteria coupled with western blot using bacterial-specific sera has also been used to identify immunogenic antigens (14–16). However, not all immunogenic proteins are expressed when bacteria are cultured *in vitro*, such as some toxins and secreted proteins. In addition, the proteins transferred to the blot membrane are denatured proteins that cannot be bound by antibodies that recognize conformational epitopes.

By focusing on *Streptococcus suis*, we aimed to develop a novel unbiased genome-wide approach to identify the highly immunogenic antigens conserved across different serotypes. *S. suis* is an emerging zoonotic pathogen that has caused three human outbreaks (17). Although most human infections have occurred in Asia, particularly in China and Southeast Asian countries, sporadic cases have been reported in 34 countries worldwide (18). Swine are considered a natural reservoir for *S. suis*, and almost all reported human cases of *S. suis* infection have had close contact with pigs or raw pork (19–22). Although it is not clear whether human-to-human transmission occurs through direct contact with patient materials, recent studies suggest that the risk of human *S. suis* outbreaks is increasing (23). In addition, numerous antibiotic-resistance genes have been found in human-associated clade (HAC) strains (23), highlighting the urgent need for effective vaccines to prevent *S. suis* infections. Currently, only inactivated vaccines are available for animal use, and their protective efficacy is serotype- or strain-dependent (24). The identification of antigenic proteins that are conserved across different serotypes is the first step for the development of broadly effective *S. suis* vaccines. Many immunogenic proteins of *S. suis* have been identified, but many of these are not core genes and are absent in some serotypes or strains (24–28). Therefore, genome-wide identification of all antigenic proteins and definition of vaccine antigens will greatly accelerate the development of universal *S. suis* vaccines for both animal and human use.

Here, we have developed an innovative genome-wide technique, BacScan, to address the challenges of bacterial vaccine development. By combining phage display, immunoprecipitation, and next-generation sequencing, BacScan overcomes traditional limitations and enables comprehensive identification of all the *S. suis* HIPs in a single assay. This breakthrough not only offers promising avenues for *S. suis* vaccine development, but also represents a significant step forward in the fight against global antimicrobial resistance.

## 2 Materials and methods

### 2.1 Ethics statement

Four- to eight-week-old female BALB/c mice were obtained from the Laboratory Animal Center of Huazhong Agricultural University, Hubei, China. All animal experimental procedures were approved by the Research Ethics Committee Huazhong Agricultural University, Hubei, China (HZAUMO-2023-0068). The studies of human sera were reviewed and approved by the Ethic Committee Tongji Medical College, Huazhong University of Science and Technology, Hubei, China (2023S080).

### 2.2 Bacterial strains and phages

*S. suis* strain SC19 was cultured in tryptic soy broth or on tryptic soy agar containing 10% newborn bovine sera. *E. coli* strains DH5α (*hsdR17(rK− mK+) sup^2^*) and BL21 (DE3) were used for plasmid construction and protein expression, respectively. T7 phages were used to construct the phage library displaying proteins of *S. suis* strain SC19 as described below (Construction of T7 phage display library). *E. coli* BLT5615 was used as host cells for the propagation of T7 display library.

### 2.3 Serum samples

Six-week-old female BALB/c mice were obtained from the Laboratory Animal Center of Huazhong Agricultural University, Hubei, China, for the preparation of *S. suis* hyperimmune sera. Mice were randomly divided into two groups. Mice in group one (n=5) were infected intraperitoneally with 4×10^7^ CFU *S. suis* SC19 strain three times on days 0, 14, and 21. Mice in group two (n=5) received 200 μL PBS and were used as negative controls. Blood samples were collected from the tail vein 7 days after the third infection. Clinical pig sera (n=20) and *S. suis* negative pig sera (n=20) were obtained from the Animal Disease Diagnostic Center of Huazhong Agricultural University, Hubei, China. Sixty human sera were collected from active swine farm workers with varying lengths of history of close contact with pigs. Of these, 46 had a history of close contact with pigs for >5 years and 14 had ≤5 years of exposure. At the time of serum collection, none of them had any symptoms of infection, and it was unclear whether they had been previously infected with *S. suis*. Twenty serum samples from children with no history of close contact with pigs were used as controls.

### 2.4 Construction of T7 phage display library

The core genes of *S. suis* were identified as described previously (29). Briefly, all annotated coding sequences (CDSs) from the *S. suis* SC19 genome were used as queries in BLASTN searches against a nucleotide BLAST database of all *S. suis* complete genome sequences. Core genes were defined as those with >80% identity over at least 80% of the length of the equivalent SC19 CDS. The core genes were amplified by PCR using gene-specific primers from *S. suis* strain SC19 genomic DNA. The genes over 600 bp were split into 600 bp fragments with 300 bp overlap between adjacent fragments. The 600-bp fragments were used for library construction instead of full-length genes. If the last fragment of a gene is less than 600 bp, the fragment was extended to 600 bp towards to the 5’ end of the gene. For the genes smaller than 600 bp, the fragment was extended to 600 bp towards to the next gene. The PCR products were digested with EcoRI/HindIII or BamHI/NotI depending on the presence of endonuclease recognition sites in the genes. Generated DNA fragments were then ligated into the T7 10-3b vector (Millipore) linearized with the same endonucleases to generate T7 gp10B-*S. suis* fragment fusion genes. The ligation products were packaged into T7 phage capsid *in vitro* using T7 packaging extracts and the generated recombinant T7 phages were propagated in *E. coli* BLT5615.

### 2.5 Phage immunoprecipitation

Phage immunoprecipitation was performed as described previously (30). Briefly, 1.5 mL microcentrifuge tubes were blocked with 3% BSA at 4°C overnight. Mouse serum containing 2 μg IgG and 2.1×10^8^ pfu phages was mixed in 1.5 mL microcentrifuge tube. After 18 h incubation at 4°C with rotation, 40 μl of protein A/G magnetic beads (Thermo Fisher Scientific) were added and further incubated for 4 h at 4°C. The mixture was centrifuged at 900×g for 1 minute at room temperature, and the tube was then placed onto a magnetic stand to remove the supernatant. The magnetic beads were resuspended with 600 μL IP wash buffer (50 mM Tris- HCl, pH 7.5, 150 mM NaCl, 0.1% NP-40) and transferred to a new 1.5 mL microcentrifuge tube. After two washes with 1 mL of IP wash buffer, the magnetic beads were resuspended with 40 μL deionized water, and the coprecipitated phages were lysed at 98°C for 10 minutes. Mouse IgG concentration was determined using the bovine serum albumin (BSA) as a standard. All samples were performed in triplicate.

### 2.6 Phage PCR and sequencing

The *S. suis* gene fragments were amplified from phage lysis using the primers F1 (5’-TTGTCTTCCTAAGACCGCTTGGCCTCCGACTTGGGGTTAACTAGTTACTCGAGTGCGG-3’) and R1 (5’-CCGAACGCAGCAAACTACGC-3’), which are phage specific and bind to the upstream and downstream of the *S. suis* gene fragment, respectively. The generated PCR products were used as templates for the second round PCR using primers F2 (5’-GAACGACATGGCTACGATCCGACTTTCGTATTCCAGTCAGGTGTGATGCTCGG-3’) and R2 (5’-TGTGAGCCAAGGAGTTGxxxxxxxxxxTTGTCTTCCTAAGACCGCTTGGCC.

T-3’, the “xxxxxxxxxx” represents 10 nt MGI regular index sequence to distinguish different samples), which are also phage specific and bind to the upstream and downstream of the *S. suis* gene fragment, respectively. PCR products were purified by agarose gel electrophoresis and sequenced on a MGISEQ-2000 NextSeq platform (BGI) at the National Key Laboratory of Crop Genetic Improvement, Huazhong Agricultural University. Low-quality sequencing data were filtered out using fastp (ver 0.20.0), and the PCR amplification-induced adapter sequences were removed using cutadapt (ver 1.18) with the default settings (31). The resulted clean data were used to identify the highly immunogenic proteins as described previously (30).

### 2.7 Bioinformatic analysis of the HIPs

The function of 793 core proteins of *S. suis* and 12 reported reference proteins were annotated in the Eggnog5 database (http://eggnog5.embl.de/#/app/home), and the members in each category were counted before and after screening. The enrichment of each category was determined by Fisher Exact Test. The virulence-associated genes (VAGs) of the *S. suis* in the 805 genes were identified by local blastp program using the VAG database described in the previous study (32). The enrichment of VAGs after screening was analyzed using Fisher Exact Test. Sequence similarity of HIPs with other proteins was determined using NCBI blastp online tool. Amino acid sequence identity of HIPs among different *S. suis* serotypes was calculated using all 66 *S. suis* complete genome sequences available in the NCBI database that have a well-defined serotype. *Streptococcal* including species commonly found in human clinics were selected to analyze the amino acid sequence identity of HIPs among genera. Extra-*Streptococcus* species were used to identify the sequence identity of *S. suis* HIPs in other bacteria. Blast results with coverage less than 80% coverage were ignored. The homology data were shown on a heatmap generated by the R package “pheatmap”, and the “Out Genus” part of the heatmap only included species homologous with at least 5 HIPs.

### 2.8 Expression and purification of recombinant HIPs

The genes encoding highly immunogenic proteins (HIPs) were amplified from the genome of strain SC19 and cloned individually into linearized pET-28a or pET-32a vector. The generated plasmid was transformed into *E. coli* BL21 (DE3), and the expression of recombinant protein was induced with 1mM isopropyl-β-D-thiogalactoside at 30°C for 4 hours. The *E. coli* cells were harvested by centrifugation at 8,000×g for 10 min at 4°C and lysed by high-pressure cell disruptor at 4°C. Cell debris was removed by centrifugation at 34,000×g at 4°C for 22 min, and the supernatant was filtered through 0.22 μm filters. The recombinant proteins were purified using a Ni-NTA column (Yeasen, Wuhan, China) and concentrated by ultrafiltration (Millipore). Protein concentration was determined by SDS-PAGE using BSA as a standard. The levels of endotoxin present in the purified proteins were determined using the Limulus Amebocyte Lysate (LAL) Endotoxin Quantitation Kit (Xiamen Bioendo Technology Co., Ltd, Xiameng China) and were in the range of 0.05-1.50 EU/mL.

### 2.9 Antibody dynamic analysis of HIPs

Eight-week-old female BALB/c mice were divided into 3 groups (n=5) and intranasally infected once with 2.7 ×10^7^, 8 ×10^7^, and 2.4 ×10^8^ CFU of *S. suis* SC19 strain, respectively. To determine the effect of age on antibody dynamics, four-week-old (n=5) and six-week-old (n=5) female BALB/c mice were intranasally infected with 8 ×10^7^ CFU of *S. suis* SC19. Sera were collected from the tail vein at different time points. Antigenic-specific IgG titer were determined by indirect ELISA as described below.

### 2.10 Enzyme-linked immunosorbent assay

Antigenic-specific antibody titers were determined by indirect ELISA as described previously (33). Briefly, the ELISA plates were coated with 100 ng of purified protein diluted in 100 μL sodium carbonate buffer (pH 9.6) at 4°C overnight, and then blocked with 3% BSA in 200 μL PBST (PBS containing 0.05% Tween-20) at 37 °C for 1 hour. After five washes with PBST, 100 μL of PBST-diluted mouse, pig, or human sera were added to each well. The plate was incubated at 37 °C for 1 hour followed by five washes with 250 μL PBST. The secondary antibodies (HRP-conjugated goat anti-mouse IgG, HRP-conjugated goat anti-pig IgG, HRP-conjugated goat anti-human IgG) were diluted 1:5000 in PBST and added to each well. After 50 minutes incubation at 37°C, the plates were washed five times with PBST. 100 μL of TMB (3,3′,5,5′-tetramethylbenzidine) substrate solution was added to each well and incubated for 5 minutes at room temperature. The reactions were stopped with 2 M H_2_SO_4_, and the plates were detected with a microplate reader at an absorbance of 450nm.

### 2.11 Mouse immunization and challenge

Six-week-old female BALB/c mice were randomized into 17 groups (n=5) and immunized intraperitoneally with either 30 μg purified protein in 100 μL PBS emulsified with an equal volume Montanide ISA-201 (SEPPIC, France) or 100 μL PBS control emulsified with 100 μL Montanide ISA-201. All the mice were boosted 14 days after the first immunization. Serum samples were collected from the tail vein two weeks after the last immunization. For challenge, fresh cultured *S. suis* SC19 was collected by centrifugation (8,000×g) for 5 min at 4°C. Cell pellets were resuspended with PBS. Each mouse was infected intraperitoneally with 6×10^9^ CFU of *S. suis* SC19. All mice were continuously recorded for morbidity and mortality for one week.

### 2.12 Statistical analysis

Statistical analysis was performed by Student t-test using Prism Graphpad software, and multi-group comparisons were evaluated by one-way analysis of variance (ANOVA). In all cases, p<0.05 was considered statistically significant.

## 3 Results

### 3.1 A BacScan platform for the unbiased and genome-wide screening of *S. suis* highly immunogenic proteins

**Figure 1:**
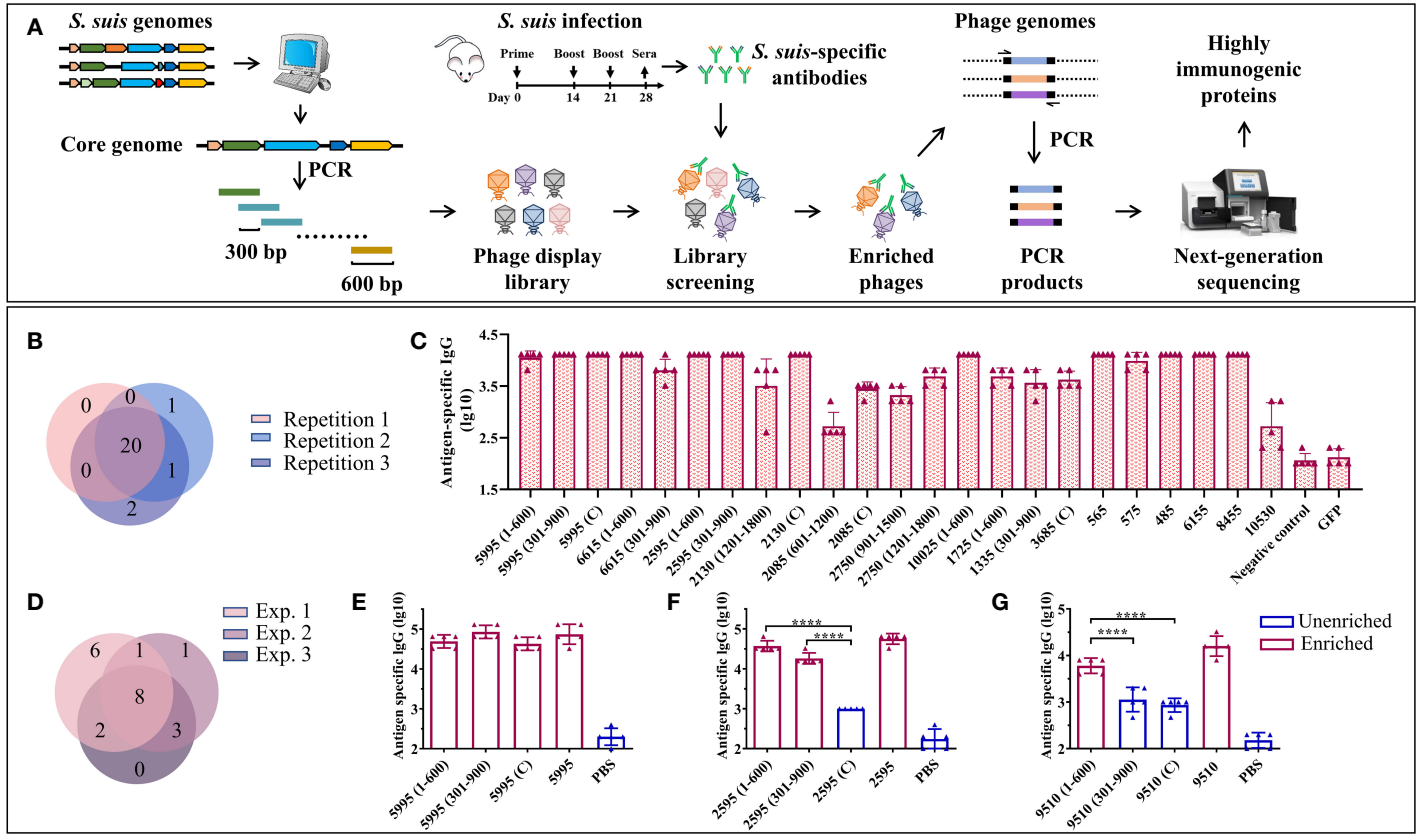
A BacScan platform for screening HIPs of *S. suis*. **(A)** Schematic diagram of BacScan for screening of HIPs from *S. suis* core genes. The *S. suis* core genes were identified using bioinformatic analysis of *S. suis* genomes and split into fragments of 600 bp in length, with 300 bp overlap between adjacent fragments by PCR. The fragments were inserted into a T7 vector to generate a T7 phage display library, which was incubated with *S. suis* specific sera. Phages bound by *S. suis*-specific antibodies were captured using protein A/G beads. The *S. suis* gene fragments in the T7 phage genome were amplified and sequenced by next-generation sequencing. The HIPs were identified by the phip-stat program. **(B)** The Venn diagram shows the screening results of three parallel experiments. A total of 24 fragments were identified, and 20 of them were enriched in three parallel experiments. The number in parentheses indicates the position of the fragment in the gene. **(C)** ELISA results show that 23 enriched fragments were recognized by *S. suis*-specific sera. The GFP protein and *S. suis*-negative mouse sera were used as controls. **(D)** The Venn diagram shows the screening results of three independent experiments using the same serum sample. **(E-G)** Identification of the highly immunogenic regions of proteins 5995 **(E)**, 2595 **(F)**, and 9510 **(G)** by BacScan, data were shown as means ± S.D. **** indicated p < 0.0001 (ANOVA).

The detailed experimental scheme of BacScan was shown in **Figure 1A**. To target antigenic proteins present in all *S. suis* strains, we used 793 previously identified core genes (29), along with 12 genes of known antigenic proteins, to construct a T7 phage library. All the 805 genes were split into 2,060 fragments of 600 bp with 300 bp overlap. These fragments were amplified by PCR using genomic DNA from *S. suis* SC19 strain, which was isolated from a piglet during the 2005 *S. suis* outbreak in Sichuan, China. The PCR products were pooled and then inserted into T7 phage genomic DNA, which was packaged into infectious phages using *in vitro* phage packaging technology to generate a phage display library. This library was incubated with *S. suis*-specific sera to capture antibody-bound phages with protein A/G coated beads. After multiple washes to remove the unbound phages, the gene fragments from the enriched T7 phages were amplified by PCR using a pair of phage-specific primers and sequenced by next-generation sequencing (NGS). The 600-bp fragments were used instead of full-length genes because homogeneous-length fragments can significantly reduce the bias of PCR amplification. In addition, since the average size of the protein structural domain is about 200 amino acids (34), we hypothesized that 600-bp fragments could preserve the protein structure intact to the greatest extent. After NGS sequencing, all the HIPs can be identified by analyzing the read count for each fragment before and after enrichment using the model described in VirScan (35).

Ideally, *S. suis* positive sera used for screening should be prepared using specific pathogen-free (SPF) pigs. This minimizes the risk of antibody cross-reactivity between *S. suis* and the other bacteria, which is a potential issue when using clinical *S. suis*-positive pig sera that may contain antibodies against other bacteria. However, the use of SPF pigs is prohibitively expensive. As a starting point, we used mouse sera and confirmed the identified antigens using clinical pig sera. The *S. suis*-specific mouse sera were prepared by infecting SPF mice three times by intraperitoneal (i.p.) injection of 4×10^7^ CFU *S. suis* SC19 strain, and sera were collected to confirm the presence of *S. suis*-specific IgG by ELISA (**Figure 1A**, **Supplementary Figure S1A**).

Approximately 2 μg of IgG per sample was used to enrich phages displaying *S. suis* immunogenic proteins. Each sample was run in triplicate to assess the reproducibility of the BacScan. A total of 24 gene fragments belonging to 17 genes were identified, 20 of which were enriched in three parallel experiments (**Figure 1B**), demonstrating the reproducibility of the BacScan. Among these potential HIPs, 2750, 5995, 10025, and 6615, have been reported in previous studies (36–39). We attempted to express all of these gene fragments in *E. coli* and were able to purify 23 recombinant proteins. Since the 10530 fragment (301–900) tends to aggregate during purification, we expressed the full-length 10530 protein instead of the fragment. The binding activities of these proteins to *S. suis*-specific sera were determined using ELISA (**Figure 1C**, **Supplementary Figure S1B**). The GFP protein and negative mouse sera were used as controls. All the 23 recombinant proteins but not the GFP control can be recognized by *S. suis*-specific sera (**Figure 1C**). However, the IgG endpoint titers of different proteins/fragments are variable. Titers of anti-2085 (601–1200) and anti-10530 IgG are lower than other HIPs specific IgG. (**Figure 1C**). Four fragments 1335 (301–900), 2750 (901–1500), 6615 (301–900), and 1725 (1–600), which were enriched in only one or two of the three parallel experiments, can also be recognized by *S. suis*-specific sera. To avoid missing HIPs, we considered the fragments as long as they were enriched in at least one of the three parallel experiments.

To determine inter-experimental variation, BacScan was performed on the same serum sample in two additional independent experiments (**Supplementary Figure S2**). A total of 21 HIPs were identified (**Figure 1D**, **Supplementary Table S1**), 9 were shared between experiments 1 and 2, 10 were shared between experiments 1 and 3, 11 were shared between experiments 2 and 3, and 8 were shared in all three experiments. In addition, each of the independent experiments was performed in triplicate, and most of the HIPs were identified in three parallel replicates (**Supplementary Figures S2A–C**), further demonstrating the reproducibility of the BacScan. These results suggest that there is variation in the BacScan between independent experiments. However, the number of HIPs identified for the same serum sample became increasingly saturated as the number of screens increased, and few new antigens were identified after three independent experiments (**Supplementary Figure S2E**).

In addition, one of the most significant advances presented by BacScan is its ability to pinpoint the highly immunogenic regions within proteins (**Figures 1E–G**), which represents a significant advancement in antigen discovery. For instance, the immunogenic proteins 5995, 2595, and 9510 were all split into three fragments in the T7 library, and three out of nine fragments were not enriched in our BacScan screen (**Figures 1E–G**). We expressed all the 9 fragments as well as the full-length proteins to compare their binding activities to *S. suis*-specific sera using ELISA (**Figures 1E–G**, **Supplementary Figures S1B**, **S2F**). We found that the endpoint IgG titers of the enriched fragments were significantly higher than those of the unenriched fragments (**Figures 1E–G**), indicating their heightened immunogenicity. To reduce the workload, we use the full-length protein instead of the enriched fragments, unless otherwise indicated.

### 3.2 Characterization of *S. suis* highly immunogenic proteins

**Figure 2:**
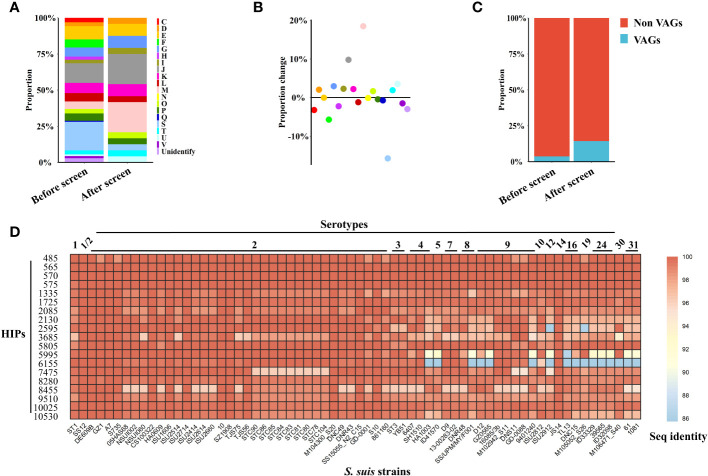
Characteristics of *S. suis* HIPs and their homology analysis. **(A, B)** Proteins related to the cell wall/membrane/envelope biogenesis were significantly enriched (p=0.0037, Fisher exact test). **(A)** The 805 proteins contained in the phage library were assigned to 20 annotated COGs, and the proportion of each COG before and after screening was shown. **(B)** The proportional changes of each COG before and after screening. **(C)** The proportion of VAGs is significantly increased after BacScan screening (p=0.0466, Fisher exact test). **(D)** Heatmap showing the amino acid sequence identity of 19 *S. suis* HIPs among different serotypes. The sequence identity of HIPs among different *S. suis* serotypes was calculated using all 66 *S. suis* complete genome sequences available in the NCBI database that have a well-defined serotype. The *S. suis* SC19 strain was used as a reference.

To determine the characteristics of *S. suis* HIPs, we first performed Clusters of Orthologous Groups (COGs) analysis of the 805 proteins included in our phage library and obtained 20 annotated COGs (**Figure 2A**, before screen). The 21 HIPs identified in the current study were categorized into 13 COGs (**Figure 2A**, after screen). In particular, the cluster M, which is related to the cell wall/membrane/envelope biogenesis, was significantly overrepresented (p=0.0037, Fisher exact test). The proportion of cluster M increased from 5.3% pre-screen to 23.8% post-screen (**Figure 2B**, pink dot). This could be explained by the fact that the surface-exposed proteins are more likely to be recognized by the immune system and therefore have a higher chance of being captured by *S. suis*-specific sera. Indeed, surface exposure is a key criterion of reverse vaccinology to predict vaccine antigens (40–44), and surface-exposed proteins were significantly enriched in known bacterial vaccine antigens (45). The COG cluster S, whose function is unknown, was negatively associated with the HIPs (**Figures 2A, B**).

Antibodies directed against virulence factors can neutralize bacterial virulence and provide protection against infection. Therefore, virulence factors may be good vaccine targets. Previous studies have identified 71 virulence-associated genes (VAGs) from the *S. suis* pan-genome (32), and 32 of them are core genes and were included in our phage library. Interestingly, we found that the proportion of *S. suis* virulence-associated proteins were significantly enriched in the HIPs (p= 0.0466, Fisher exact test), increasing from 4.0% to 14.3% (**Figure 2C**).

The conserved antigens across different serotypes could be good targets for the development of broadly protective bacterial vaccines. Of the total 21 HIPs identified, two previously reported HIPs, 6615 and 2750, are not encoded by core genes and were therefore excluded from the subsequent sequence analysis. We found that all 19 HIPs encoded by core genes are highly conserved among different *S. suis* serotypes with 86-100% sequence identity (**Figure 2D**), suggesting that they could be targets for the development of broadly protective *S. suis* vaccines. 15 HIPs also share 74%-99% sequence identity with proteins from other bacteria within or outside the genus *Streptococcus* (**Supplementary Figure S3**). Interestingly, two HIPs, 2085 and 6155, are highly conserved across serotypes but have relatively low homology with proteins from other bacteria (with less than 62% sequence identity) (**Figure 2D**, **Supplementary Figure S3**), suggesting that they may be specific targets for the development of serological techniques for *S. suis*.

### 3.3 Dynamic analysis of antibody repertoires against HIPs revealed diagnostic targets for serological detection of *S. suis*

**Figure 3:**
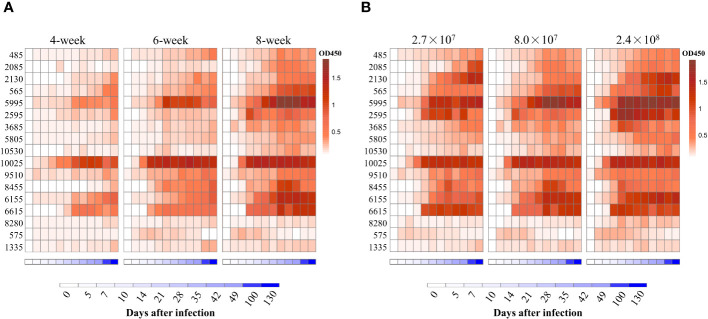
Dynamic analysis of antibody repertoires against HIPs after single infection with *S. suis* strain SC19 in mice. **(A)** Dynamic analysis of antibody repertoires in mice of different ages. Four- to eight-week-old mice were infected intranasally with 8.0×10^7^ CFU *S. suis* strain SC19. **(B)** Dynamic analysis of antibody repertoires in mice infected with different doses of *S. suis* SC19 strain. Eight-week-old mice were infected intranasally with 2.7×10^7^ (low dose), 8.0×10^7^ (medium dose), and 2.4×10^8^ (high dose) CFU *S. suis* SC19 strain, respectively. Sera were collected at different time points to determine HIP-specific IgG titers. Each row represents one HIP, and each column indicates the time after infection. The red color scale represents the O.D_450_ value of the ELISA. The blue color scale represents the time points.

To define which HIPs could be targets for vaccine development and serological diagnostics, we first determined the kinetics of antibody responses to the HIPs after *S. suis* infection. Four- to eight-week-old mice were infected once by intranasal (i.n.) administration of 8.0×10^7^ CFU *S. suis* SC19 strain, and sera were collected at different time points to determine HIP-specific IgG using ELISA. We attempted to express all the 21 HIPs in full-length using *E. coli* BL21, but failed to obtain the 1725, 570, 7475, and 2750 due to either solubility issues or weak binding to the HisTrap column (**Supplementary Figures S1B, S2F, S4**). The resulting 17 recombinant proteins were used individually as ELISA coating antigens. Most of the HIPs were able to induce antigen-specific IgG approximately 14 days after infection and persisted for at least 130 days (**Figure 3A**). Overall, the reactivity of sera to HIPs increased with age. The 4-week-old mice were less efficient in producing HIP-specific IgG antibodies than the 6-week-old and 8-week-old mice, which may be due to their immature immune systems. Although we were able to detect antibodies against all 17 HIPs, there was variation among the HIPs. We found that IgG antibodies were mainly directed against 13 HIPs, especially proteins 5995 and 10025 (**Figure 3A**). The 5995-specific IgG was detected at 10 days and peaked at 21 days post infection. Similarly, the 10025-specific IgG was peaked at 10 days post infection (**Figure 3A**). To determine whether the dose of infection affected the kinetics of antibody responses, 8-week-old mice were infected with 2.7×10^7^ (low), 8.0×10^7^ (medium), and 2.4×10^8^ (high) CFU *S. suis* SC19 strain, respectively. The dynamics of IgG antibody responses to the HIPs were similar between the medium- and high-dose groups. However, the development of HIP-specific IgG antibodies was delayed by 2-3 days in the low-dose group. Again, IgG antibodies were mainly directed against 13 HIPs, especially proteins 5995 and 10025 regardless of the infection dose (**Figure 3B**).

**Figure 4:**
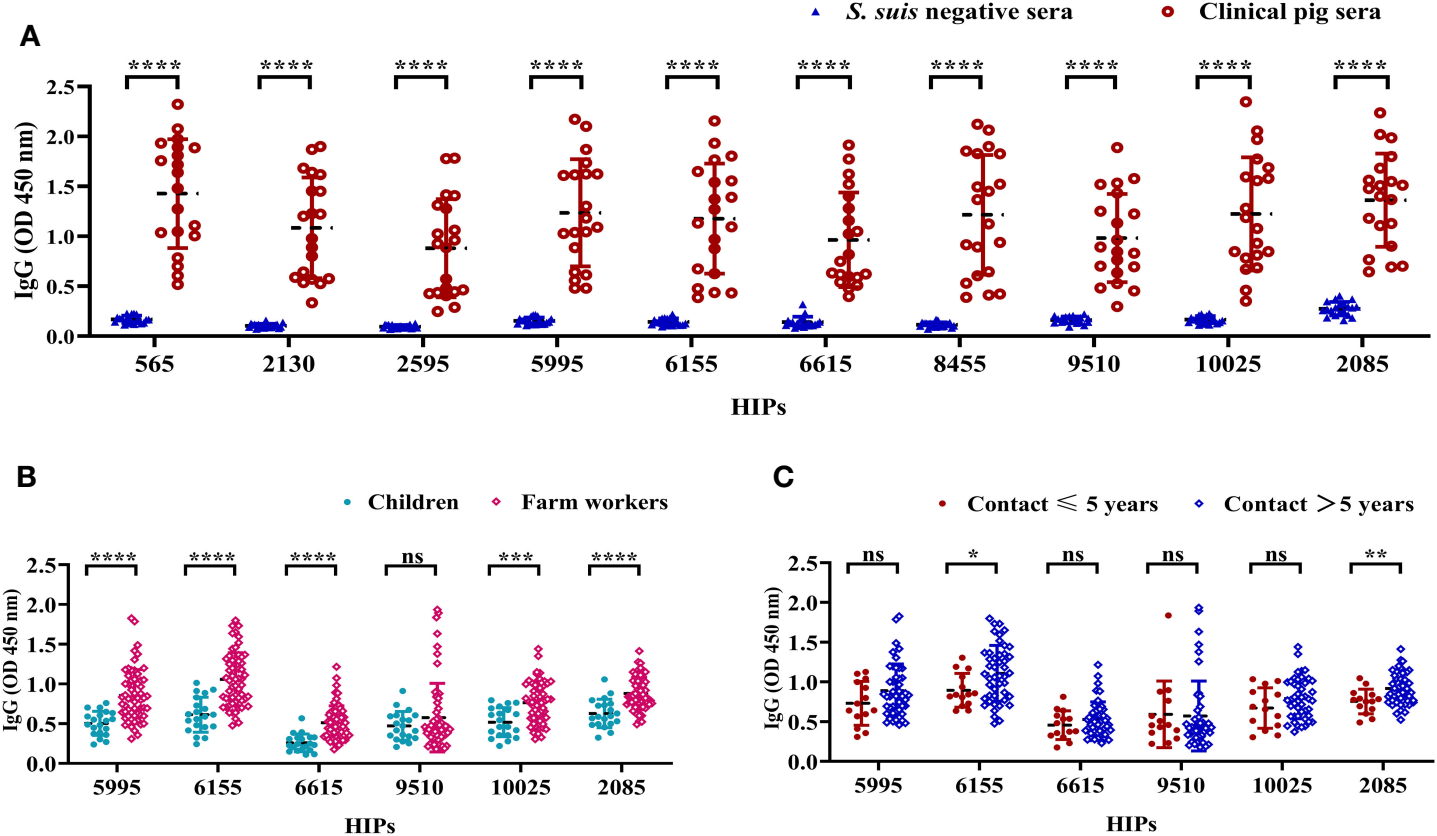
The reactivities of selected HIPs to swine and human sera. **(A)** The binding activities of HIPs to pig sera. Twenty clinical pig sera and 20 *S. suis*-negative sera were used to determine HIP-specific IgG by ELISA. Data were represented as mean ± S.D. ****P < 0.0001 (Student’s t-test). **(B)** ELISA results showing the binding activities of six HIPs to human sera. Sera from farm workers with a history of close contact with pigs (n=60) showed higher O.D_450_ than sera from children with no history of contact with pigs (n=20) for all tested HIPs except 9510. **(C)** Binding activities of six HIPs to sera from humans with varying histories of exposure to pigs. HIPs 6155 and 2085 have higher binding activities to sera from humans with a history of > 5 years of close contact with pigs (n=46) compared to sera from humans with ≤ 5 years of history of exposure to pigs (n=14). Data were presented as mean ± S.D. *P < 0.05; **P < 0.01; ***P < 0.001; ****P < 0.0001 (Student’s t-test).

We then focused on the top 10 HIPs that induced high IgG titers. To further determine the reactivities of pig sera to these HIPs, 20 clinical pig sera and 20 *S. suis*-negative sera were collected for ELISA analysis. As shown in **Figure 4A**, all the 10 HIPs can be recognized by clinical pig sera, although they exhibit variable binding activities. These results indicated that these pigs were likely infected with *S. suis* and further confirmed that these HIPs are highly immunogenic in pigs. However, since 6 of these HIPs showed high sequence identity (74-97%) to the proteins in other bacteria such as *E. coli*, *Salmonella enterica*, and *Staphylococcus aureus* (**Supplementary Figure S3**), we cannot exclude the possibility that these pigs were infected with other bacteria and that the ELISA signals were due to the cross-reactivity of the sera. As expected, *S. suis* negative sera did not react with these HIPs (**Figure 4A**). Significantly, HIPs 2085 and 6155 which have low homology to proteins from other bacteria, showed high reactivities with clinical sera. In particular, the lowest value of O.D_450_ value in the clinical group is 4 times higher than that of the negative control for HIP 2085, making it an attractive target for the development of serological diagnostic methods.

Individuals with a history of close contact with pigs may be at higher risk for *S. suis* infection. To determine whether HIPs 2085 and 6155 could discriminate potential human infection, sera were collected for ELISA analysis from 60 farm workers with a history of close contact with pigs for 1-20 years. Sera from children with no history of contact with pigs were used as controls. HIPs 5995, 6615, 9510, and 10025 were also included in the serologic analysis. The ELISA results showed that the sera from farm workers gave significantly higher O.D_450_ than the control sera for all tested HIPs except 9510 (**Figure 4B**). Interestingly, the 2085- and 6155-specific O.D_450_ are significantly different between workers with more than 5 years and less than 5 years of experience in pig farming (**Figure 4C**). These results suggest that farm workers are at higher risk of contracting *S. suis* infection and that the risk of infection increases with the number of years of experience. HIPs 2085 and 6155 may be good targets for *S. suis* serological diagnostics in humans. However, more experiments are needed to further evaluate their potential.

### 3.4 Identification of conserved protective antigens for the development of broadly protective subunit vaccines against *S. suis*

**Figure 5:**
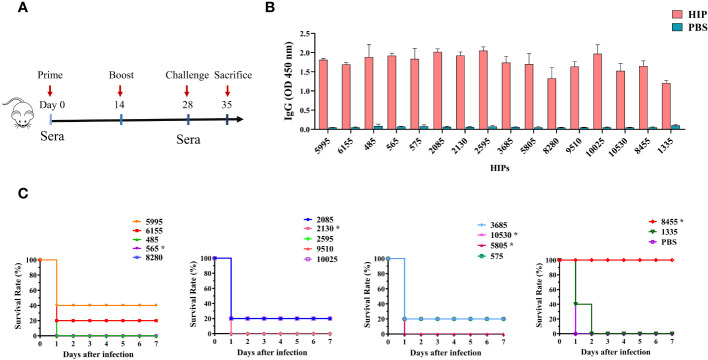
Immunogenicity and protective efficacy of HIPs in mice. **(A)** Mice were immunized twice by intraperitoneal injection, and sera were collected according to the scheme. **(B)** HIPs-specific IgG antibody titers were determined by ELISA. **(C)** The immune protection of each HIP was determined by challenging the mice with 6.0×10^9^ CFU *S. suis* strain SC19. Eight HIPs provide different levels of protection against challenge. Asterisks indicate that the HIPs are toxic and caused the death of the mice during boost immunization (see **Supplementary Figure S5** for the details).

Three of the 21 HIPs identified, namely 2750 (known as Ide*_Ssuis_*), 6615 (known as SAO), and 5995 (known as PrsA), have been shown to induce protective immune responses in previous studies (36–38). We randomly selected 5995 as a positive control in our animal experiment. Due to the low yield of recombinant protein expressed in *E. coli*, HIPs 1725, 570, and 7475 were also not included in the animal experiments. To define new HIPs capable of inducing protective immune responses for vaccine development, all the 16 recombinant HIPs were individually formulated with ISA 201 adjuvant and administered intraperitoneally to the mice as shown in **Figure 5A**. Sera were collected on days 0 and 28 for IgG titration, and mice were challenged with 6×10^9^ CFU *S. suis* SC19 strain 14 days after the boost. As shown in **Figure 5B**, all 16 HIPs can induce antigen-specific IgG antibodies, whereas lower titers were observed for 1335-specific IgG. Mouse sera from the PBS control group did not react with any of these HIPs.

Challenge data showed that 7 of these HIPs, namely 5995, 6155, 2085, 575, 3685, 10025, and 10530 can provide partial protection (20%-40%) to immunized mice against challenge with the highly virulent *S. suis* SC19 strain (**Figure 5C**). The *S. suis* SC19 strain was used because it belongs to the most prevalent and virulent serotype 2 and has been widely used as a standard strain for virulence studies (46). Significantly, HIP 8455 can provide complete protection against challenge compared to other HIPs. None of the remaining HIPs protected mice from challenge, although they were effective in inducing IgG antibodies. In a previous study, HIP 10025 in combination with 5 other *S. suis* proteins was tested as a vaccine candidate in pigs, but no significant protection was observed after intranasal challenge with *S. suis* serotype 14 (39). The HIP 5995 (known as PrsA), which was shown to provide 50%-66% protection against challenge with *S. suis* type 2 or 9 strains in the previous study (38), induced 40% immune protection in our study. The difference in efficacy of these HIPs observed in the current study may be due to differences in challenge dose and bacterial strain. In fact, strain SC19 is a hypervirulent strain that causes streptococcal toxic shock-like syndrome in humans, mice, and pigs (46, 47). Our results indicated that 8 HIPs are protective antigens of *S. suis* and can be used individually or in combination for the development of *S. suis* vaccines, particularly 8455. Since the purpose of the current study was to develop a novel unbiased genome-wide approach to discover the HIPs conserved across different serotypes, we did not systematically evaluate the protective efficacy of HIP 8455 against different *S. suis* serotypes using different animal models. This will be done in our future studies. In addition, we found that HIPs 565, 2130, 5805, 10530, and 8455 were toxic and caused death in mice during boost immunization (**Supplementary Figure S5**). Therefore, further work is needed to eliminate the toxicity of 8455 while maintaining its immunogenicity.

## 4 Discussion

Identification of the conserved protective antigens across different serotypes is a critical step in the development of a broadly effective bacterial vaccine. Here, we have developed a novel unbiased genome-wide approach, called BacScan, to identify the HIPs conserved across different serotypes by combining phage display, phage-immunoprecipitation, phage-PCR, and next-generation sequencing. A number of features make BacScan one of the most powerful approaches for identifying conserved immunogenic proteins for vaccine or diagnostic purposes.

First, BacScan is an unbiased genome-wide screening approach. The genome-wide display library was constructed using T7 instead of filamentous phage, which assembles in the periplasm of *E. coli* and displays only the peptides/proteins that are transported to the periplasm (12, 13). This is not the case for the lytic T7 phage, which assembles in the cytoplasm (34). Most importantly, BacScan requires only one round of panning instead of the multiple rounds of panning required by traditional phage selection (48, 49). This is important because the expression of certain exogenous proteins can limit the proliferation of the recombinant phages. As a result, these phages are lost after multiple rounds of amplification. In addition, all *S. suis* genes are split into equal length 600 bp fragments to minimize amplification bias during PCR amplification of the enriched phages (see Materials and Methods for the details).

Second, BacScan is not limited by the temporal expression of bacterial proteins. The expression of bacterial proteins is highly regulated, and not all immunogenic proteins are expressed when bacteria are cultured *in vitro* (50). Unlike some proteomics methods that use bacteria cultured *in vitro* to identify bacterial immunogenic proteins, BacScan used T7 phage to heterologously express and display all the core proteins of the pathogenic bacterium in *E. coli*. In addition, each protein was split into 200-aa fragments to avoid the possible expression issues with the full-length proteins, and therefore each protein has a higher chance of expressing at least one fragment. Therefore, all the bacterial proteins included in the T7 phage library were expected to be expressed.

Third, BacScan is a cost-effective method. Protein array is a high-throughput technology that allows for the identification of bacterial antigenic proteins (51, 52). However, the production of thousands of proteins to prepare protein arrays is very expensive, which is a limitation for some research laboratories. Reverse vaccinology reduces the number of bacterial candidate proteins, but still requires the expression of hundreds of proteins for their immunogenicity testing (9, 53). In contrast, BacScan does not require individual purification of bacterial proteins. Instead, the protein-encoding genes are cloned into the T7 phage genome and expressed during phage propagation, which is much less expensive. In addition, the foreign bacterial genes in the genome of the enriched T7 phages are amplified by PCR, where different barcodes can be included for multiple serum samples used for antigen screening. The PCR results can be pooled for next-generation sequencing, further reducing costs.

Various methods have been employed to identify bacterial HIPs over the years, each with its own set of advantages and limitations (9, 54, 55). However, BacScan is a comprehensive method that combines the advantages of each method. These key features collectively enhance BacScan’s ability to systematically identify a wide range of immunogenic proteins across the bacterial genome, while overcoming the limitations of each method. For example, BacScan can uncover a broader range of HIPs compared to methods that focus on specific subsets, such as surface proteins in reverse vaccinology or expressed proteins in the *In Vivo* Expression Technology (IVET) (43, 56, 57). BacScan, can identify low-abundance HIPs, an area where proteomics-based methods may struggle (58).

As a proof of concept, we used BacScan to scan the *S. suis* core genome for the HIPs. All the 19 HIPs identified are highly conserved among different *S. suis* serotypes with 86%-100% sequence identity. We found that 8 conserved HIPs can confer 20%-100% protection to immunized mice against challenge with the highly virulent *S. suis* SC19 strain. Six of them, namely 6155, 2085, 575, 3685, 10530, and 8455, have not been previously tested as vaccine antigens. Significantly, HIP 8455 can provide complete protection against challenge, suggesting that it may be a good target for the development of a broadly protective subunit vaccine. Since the purpose of the current study is to develop a novel unbiased genome-wide approach to discover the HIPs conserved across different serotypes, we did not systematically evaluate the protective efficacy of HIP 8455 against different *S. suis* serotypes using different animal models. This will be done in our future studies. Nevertheless, the HIP 8455 is highly conserved across different serotypes, indicating its broad protective potential against multiple serotypes.

Although BacScan is a powerful technology for identifying conserved immunogenic proteins, it has several limitations. First, the identification of protective antigens is highly dependent on the quality of the serum. If the serum cannot neutralize bacterial cells, it is impossible to screen protective HIPs using BacScan. Therefore, it is strongly recommended that the neutralizing activity of the serum be determined prior to screening. Second, the antibody cross-reactivity between bacterial species may interfere with the screening for HIPs from the desired bacterium. Many bacterial species share homologous proteins that have different sequence conservation and may cross-react with other bacteria-specific antibodies. This interference can be eliminated by using positive sera prepared from specific pathogen-free (SPF) animals. This is also the reason why we used *S. suis*-specific mouse sera to screen the HIPs. Third, the bacterial proteins in the T7 display library were split into 200-amino acid long fragments, which may disrupt the structure of some proteins. Therefore, the conformational epitopes may be missed. This can be overcome by using multiple T7 libraries displaying proteins of different lengths and each library displaying proteins of the same length. In addition, as long as one of its fragments has been enriched by BacScan, the full-length bacterial protein will be identified as HIP and used for vaccine development. This can minimize the effect of conformational constraints.

In summary, we have developed an unbiased and genome-wide approach, BacScan, to scan the bacterial core genome for the highly immunogenic proteins. This technology can cost-effectively identify conserved antigenic proteins across different serotypes. We believe that BacScan can be adapted to any bacterial pathogen by constructing a custom phage display library and will accelerate the development of a broadly protective bacterial subunit vaccine.

## Data availability statement

The raw data supporting the conclusions of this article will be made available by the authors, without undue reservation. The next-generation sequencing data were deposited in Sequence Read Archive (SRA) of the NCBI database with the BioProject accession number PRJNA1107140.

## Ethics statement

The studies involving humans were approved by Ethic Committee Tongji Medical College, Huazhong University of Science and Technology, Hubei, China. The studies were conducted in accordance with the local legislation and institutional requirements. Written informed consent for participation in this study was provided by the participants’ legal guardians/next of kin. The animal study was approved by Research Ethics Committee Huazhong Agricultural University, Hubei, China. The study was conducted in accordance with the local legislation and institutional requirements.

## Author contributions

JD: Conceptualization, Methodology, Writing – review & editing. QZ: Methodology, Writing – original draft. JY: Methodology, Writing – original draft. YZ: Methodology, Writing – original draft. ZM: Methodology, Writing – original draft. SP: Methodology, Writing – original draft. HQ: Methodology, Writing – original draft. CJ: Methodology, Writing – original draft. GW: Supervision, Writing – original draft. AZ: Supervision, Writing – review & editing. PT: Conceptualization, Supervision, Writing – review & editing.
